# Indications and outcome in surgically treated asymptomatic meningiomas: a single-center case-control study

**DOI:** 10.1007/s00701-020-04244-6

**Published:** 2020-02-03

**Authors:** Olivia Näslund, Thomas Skoglund, Dan Farahmand, Thomas O. Bontell, Asgeir S. Jakola

**Affiliations:** 1 Sahlgrenska Academy, Gothenburg, Sweden; 2 Institute of Physiology and Neuroscience, Sahlgrenska Academy, Gothenburg, Sweden; 3 Sahlgrenska University Hospital, Blå Stråket 7, 413 45 Gothenburg, Sweden; 4 Department of Neurosurgery, Sahlgrenska University Hospital, Gothenburg, Sweden; 5 Department of Clinical Pathology and Cytology, Sahlgrenska University Hospital, Gothenburg, Sweden; 6 Department of Neuromedicine and Movement Science, Norwegian University of Science and Technology, Trondheim, Norway

**Keywords:** Meningioma, Asymptomatic, Postsurgical complication, Outcome

## Abstract

**Background:**

Many meningiomas are detected incidentally and remain asymptomatic until intervention. The goal of this study was to describe the management and outcome in this group of surgically treated asymptomatic meningiomas.

**Methods:**

From 2004 to 2017, 45 patients with asymptomatic meningioma were surgically treated at Sahlgrenska University Hospital, and their medical records and imaging data were analyzed. The asymptomatic cases were matched with symptomatic ones with respect to age at diagnosis, location, WHO (World Health Organization) grade, and Simpson grade.

**Results:**

Time from diagnosis to surgery differed between the asymptomatic and symptomatic patients (8.6 vs. 1.3 months; *p* < 0.001). Of symptomatic patients, 32.6% still used anti-epileptic drugs > 1 year after surgery, compared with 7.7% of the asymptomatic (*p* = 0.003). Thirty-day complication rate was significantly higher among the asymptomatic cases (35.6% vs. 24.4%; 0.001), as well as the proportion of older asymptomatic individuals (> 70 years) experiencing postoperative complication compared with symptomatic patients of the same age group.

**Conclusion:**

As expected, asymptomatic cases had smaller tumors and waited longer for surgery. Surprisingly, complication rate was significantly higher among asymptomatic cases compared with their symptomatic control. Taken into account that many asymptomatic tumors are removed surgically due to patient’s wish, one might suggest a more restrictive approach, especially in the elderly.

**Electronic supplementary material:**

The online version of this article (10.1007/s00701-020-04244-6) contains supplementary material, which is available to authorized users.

## Introduction

Meningiomas are one of the most common brain tumors, accounting for 13–37% of all intracranial neoplasms [8, 10]. Meningiomas in need of treatment can be managed with surgery, fractionated radiotherapy, and radiosurgery. Epidemiological surveys have found that incidental meningiomas constitute 39% of all diagnosed meningiomas, and the incidence of asymptomatic meningioma was found to be significantly higher in individuals over the age of 70 [10]. With an ever-aging population and in an era of extensive brain imaging, the management of incidental meningiomas has become an increased clinical challenge [21]. Recent EANO (European Association of Neuro-Oncology) guidelines indicate that active monitoring is the most appropriate management strategy in most asymptomatic cases; although the guidelines give no specific recommendation on the frequency and duration of follow-up [5]. Many advocate for active monitoring during a period of 5 years, as incidental meningiomas with growth potential often show growth within this time-period [7]. It has been reported that tumor growth occurs in 75% of incidental tumors over a period of 15 years [8].

Radiological progression, development of symptoms, and patient preference have been reported as indications for treatment for incidental meningiomas [7]. In line with the reports of a 5-year active monitoring period, a recent review found that intervention was carried out within 5 years of diagnosis in 94.3% of initially asymptomatic meningiomas [7]. Hence, if radiological progression should be a reason for intervention, at least 75% of patients with initial active monitoring would eventually be candidates for surgery. However, one study found that the proportion that experienced neurological morbidity was close to 14% in asymptomatic patients with meningioma undergoing surgery [26].

Intervention at diagnosis of asymptomatic meningiomas, or intervention simply because the meningioma at some point demonstrates growth, might lead to overtreatment, which is a general concern of modern health care [2]. There is a need to further investigate the implications of current practice, to assess if and why surgery for asymptomatic patients is performed and to study the outcome, in order to properly weigh possible long-term advantages of surgery against demonstrated short-term disadvantages [7]. To guide clinical management, more detailed reports on indications and postoperative outcomes are needed.

The aim of this study was to describe in detail the management and outcome in the controversial group of surgically treated asymptomatic meningiomas, and to compare outcomes with a control group.

## Materials and methods

### Patients

The Neurosurgical Department at Sahlgrenska University Hospital is the sole provider of brain tumor surgery in the region of Western Sweden and serves a population of approximately 1.7 million. We retrospectively reviewed patients treated for brain tumors at our institution from January 2004 to December 2017. Patients undergoing first time neurosurgical intervention for a histologically verified meningioma were selected for further analysis. An asymptomatic tumor was defined as no symptoms at time of diagnosis (i.e., incidental finding), or symptoms that could not be attributed to the size or location of the tumor. In this study, we only selected patients who remained asymptomatic during the follow-up period up to surgery. Patients who had previously undergone meningioma surgery, as well as patients with missing baseline variables, were excluded.

The data extracted included age and gender, reasons for brain imaging leading to radiological diagnosis and presence of deficit at diagnosis; Karnofsky score [19] and work status at time of diagnosis; date of radiological diagnosis; location, size, Simpson grade [20]; postoperative complications, neurological deficits, histopathological grading, course of disease, and adjuvant therapy; as well as work status at > 1 year postoperatively and if the patients had deceased before end of follow-up (January 1, 2018). Focal deficit was defined as loss of vision, language, motor, or sensory functions. Recurrence was defined as new lesion in a patient with radical removal while progression was defined as growth of known remnant. Predisposing factors such as irradiation and genetic syndromes (e.g., neurofibromatosis 1, neurofibromatosis 2, Li-Fraumeni, Turcot, Gardener, Cowden, Gorlin, or multiple endocrine neoplasia type 1) were registered. The largest diameter was measured in the sagittal, coronal, or axial plane using the most recent MRI (magnetic resonance imaging) prior to surgery and the tumor volume was measured using 3D-Slicer. The location of the tumor was classified as olfactory groove, suprasellar, clivus, foramen magnum, cerebellar, parasagittal, paranasal, optic sheath, sphenoid wing, posterior fossa, tentorial, falx, convexity, and intraventricular. Tumor grade was according to the WHO (World Health Organization) grade used at time of clinical diagnosis [13, 17].

With the use of extracted data, we calculated the Asian Intracranial Scoring System (AIMSS) [11] to estimate the risk of rapid growth in untreated asymptomatic meningiomas. When using AIMSS, one takes into account tumor size, calcification of the tumor, peritumoral edema, and the signal on T2-weighted MRI. Tumor size was measured in diameter, calculated using tumor volume and the assumption that all meningiomas are perfect spheres, as done by others [11]. Then the tumors were categorized into three groups of < 2.5 cm, ≥ 2.5 to <4 cm, and ≥ 4 cm, each group awarded 0, 3, and 6 points, respectively. Absence of calcification was awarded 2 points, while presence of peritumoral edema was awarded 1 point. Signal on T2-weighted MRI was categorized into two groups of hypointense and hyper/isointense and given a score of 0 or 2 points, respectively. A total score of 0–2 points was classified as low risk, a score of 3–6 intermediate risk, and 7–11 points as high risk.

The patients were matched with a control group of symptomatic meningiomas also treated at our institution. The matching criteria were age at primary surgery, location of tumor, WHO grade, and Simpson grade. Three out of four correctly matched variables were needed to be considered a successful match. The matching was done unaware of clinical and outcome variables other than the criteria used for matching.

### Matching characteristics

Age at surgery was 56.1 years (± 11.5) in asymptomatic patients and 56.5 years (± 12.0) in controls (*p* = 0.92). Eight asymptomatic cases and 8 controls were above 70 years old. No significant difference (*p* = 1.0) was found between the two groups with regard to tumor location, as all but one case of a falcine tumor could be perfectly matched with their respective control. Also, Simpson grade and WHO grade corresponded well between the matched groups (*p* = 0.88 and *p* = 1.0 respectively) (Table 1).

**Table 1:** Matching characteristics

Matching characteristics	Case (*n* = 45)	Control (*n* = 45)	*p* value
Age at surgery, mean (SD)	56.1 (11.5)	56.4 (12.0)	0.92
Tumor location			1.0
Convexity, *n* (%)	21 (46.7)	22 (48.9)	
Parasagittal, *n* (%)	3 (6.7)	3 (6.7)	
Falx, *n* (%)	10 (22.2)	9 (20)	
Tentorium, *n* (%)	1 (2)	1 (2)	
Sphenoid wing, *n* (%)	7 (15.6)	9 (15.6)	
Suprasellar, *n* (%)	1 (2.2)	1 (2.2)	
Paranasal/olfactory, *n* (%)	2 (4.4)	2 (4.4)	
Cerebellum, *n* (%)	1 (2.2)	1 (2.2)	
WHO grade			0.88
Grade I, *n* (%)	39 (88.6)	41 (91.1)	
Grade II, *n* (%)	4 (8.9)	3 (6.7)	
Grade III, *n* (%)	1 (2.2)	1 (2.2)	
Missing, *n* (%)	1 (1.1)	0 (0)	
Simpson grade			1.0
I, *n* (%)	21 (46.7)	22 (48.9)	
II, *n* (%)	19 (42.2)	18 (40)	
III, *n* (%)	0 (0)	0 (0)	
IV, *n* (%)	5 (11.1)	5 (11.1)	

### Systematic literature search of the field

Librarians at the Medical Library at Sahlgrenska University Hospital performed a systematic search of literature on the topic. The search was conducted 1st of July 2019 in PubMed and rendered 180 articles published between 1995 and 2019. Accepted languages were English and Scandinavian languages. The search used keywords “meningioma,” “asymptomatic,” and “surgery” as well as their synonyms. There were 30 articles identified from this literature search based on their relevance to this topic.

### Statistical analyses

Statistical analyses were performed using IBM SPSS 25 software. A *p* value of < 0.05 was considered significant. All tests were two-sided, and central tendencies were presented as means ± SD or median and first and third quartile if skewed. Normality was assessed using Kolmogorov–Smirnov test. Continuous data was analyzed using independent sample *t* test or Mann-Whitney *U* test as appropriate. Similarly, categorical variables were analyzed using Pearson’s chi-square or Fisher exact test.

## Results

### Patient selection

**Fig. 1:**
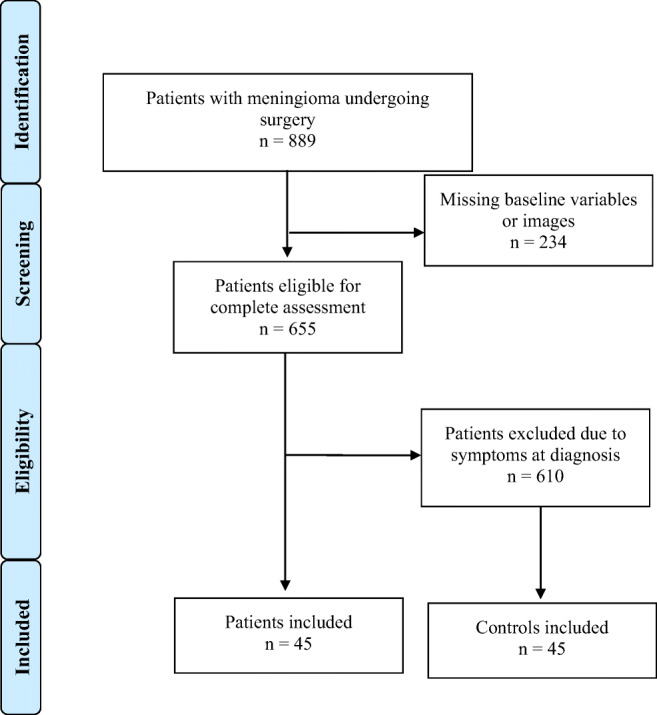
Resulting flowchart of search strategy

Figure 1 describes the patient selection process. We included 45 patients with asymptomatic meningioma and 45 controls.

### Baseline characteristics

For baseline characteristics in asymptomatic patients and controls, see Table 2*.* There was no difference in work status at time of diagnosis between asymptomatic and symptomatic patients (*p* = 0.69). There were five (11.1%) asymptomatic patients compared with one (2.2%) control patient with predisposing factors such as brain irradiation and neurofibromatosis (NF) 2.

**Table 2:** Baseline characteristics of 45 asymptomatic patients and 45 symptomatic controls treated for meningioma between 2004 and 2017

Patient and tumor characteristics	Case (*n* = 45)	Control (*n* = 45)	*p* value
Female, *n* (%)	31 (68.9)	35 (77.8)	0.48
Karnofsky score > 70, *n* (%)	41 (91.1)	41 (91.1)	1.0
Predisposing factors*	5 (11.1)	1 (2.2)	0.20
Work status			0.69
Pension, *n* (%)	15 (33.3)	15 (33.3)	
Sick leave, *n* (%)	3 (7.3)	1 (2.2)	
Partial sick leave, *n* (%)	3 (7.3)	1 (2.2)	
Working full hours, *n* (%)	19 (46.3)	24 (57.1)	
Other, *n* (%)	1 (2.2)	1 (2.2)	
Missing, *n* (%)	4 (9.8)	3 (7.3)	
Main indication(s) of surgery**			< 0.001
Symptoms, *n* (%)	0 (0)	45 (100)	
Growth, *n* (%)	15 (34.9)	4 (9.3)	
Size, *n* (%)	10 (23.3)	16 (37.2)	
Patient’s wish, *n* (%)	11 (25.6)	3 (7)	
Expected natural course of disease, *n* (%)	4 (9.3)	2 (4.7)	
Edema, *n* (%)	3 (7)	2 (4.7)	
Unknown, *n* (%)	2 (4.7)	2 (4.7)	
Preoperative embolization, *n* (%)	2 (4.4)	3 (6.7)	1.0
Preoperative AED, *n* (%)	0 (0)	16 (35.5)	< 0.001
Contact or invasion of venous sinus, *n* (%)	16 (35.6)	20 (44.4)	0.74
Mainly left-sided tumor, *n* (%)	26 (57.8)	23 (51.1)	0.53
Both sides (if multiple resected at the same time), *n* (%)	1 (2.2)	0 (0)	
Largest diameter at diagnosis in mm, mean (SD)	27.8 (12.8)	46.3 (15.3)	< 0.001
Tumor volume (cm^3^) at diagnosis, median (Q1–Q3)	9.3 (5.3–22.2)	35.7 (15.5–61.5)	< 0.001
Tumor volume (cm^3^) at surgery, median (Q1–Q3), *n* = 19	10.7 (5.7–21.2)***	N.A****	
Solitary meningioma, *n* (%)	42 (93.3)	42 (93.3)	1.0
Edema, *n* (%)	18 (40.0)	30 (66.7)	0.02
Calcification, *n* (%)	8 (17.8)	10 (22.2)	0.79
Hyperintensity on T2, *n* (%)	6 (13.3)	7 (15.6)	1.0

*Brain irradiation and genetic mutations such as NF2

**May exceed 100% since several indications for surgery may co-exist

***Not all cases had several MRI before surgery

****Too few controls had follow-up MRI before surgery due to short time from diagnosis to surgery

**Fig. 2:**
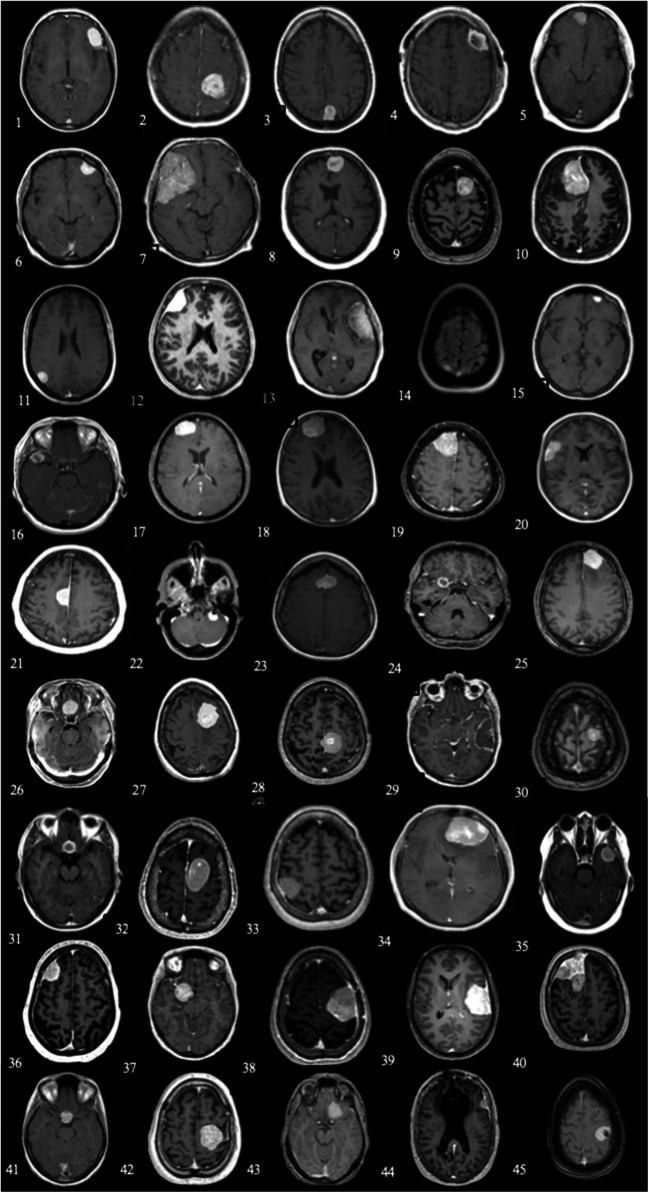
Representative T1-GD axial slice from the diagnostic MRI examination and cause of first radiological scan among asymptomatic patients. Left to right, patient 1 (upper left) through patient 45 (lower right). 1. Infarction^D^, 2. migraine, 3. idiopathic intracranial hypertension, 4. trauma, 5. depression, 6. vertigo*, 7. control of head/neck cancer*, 8. numbness in hand and arm*, 9. pulsating sensation in ear^S^, 10. fainting, 11. meningitis, 12. migraine*, 13. neck pain^S^, 14. examination before pregnancy*, 15. vertigo, 16. idiopathic intracranial hypertension*, 17. shaking in thumb caused by exertion, 18. control patient in study, 19. infarction*, 20. neck pain, 21. hallucinations, 22. vertigo*, 23. pain behind eye*^S^, 24. neck pain*, 25. infection of unspecified origin, 26. control of head/neck cancer*, 27. trauma, 28. trauma^D^, 29. control of head/neck cancer, 30. migraine*, 31. migraine*, 32. sepsis*^D^, 33. neck pain*, 34. examination of vestibula schwannoma, 35. neck pain*, 36. periorbital lipoma*, 37. trauma*^D^, 38. transient hearing loss*, 39. migraine*, 40. unspecified symptoms from ear*, 41. meningitis, 42. transient hearing loss, 43. control head/neck cancer*, 44. follow-up exam oligodendroglioma*, 45. fatigue*. The asterisk indicates growth prior to surgery. D indicates new onset deficit postoperatively. S indicates new onset seizure postoperatively

The most common indications for brain imaging among asymptomatic patients were neck pain (11.1%), migraine (11.1%), trauma (8.9%), control of head/neck cancer (8.9%), and vertigo (6.7%). The causes of first radiological scan were all deemed unrelated to the meningiomas discovered (Fig. 2).

Tumor diameter at diagnosis differed significantly between the two groups (27.8 vs. 46.3 mm; *p* < 0.001), and likewise, tumor volume at diagnosis differed significantly (9.3 cm^3^ vs. 35.7 cm^3^, *p* < 0.001). In line with the strategy of active monitoring, 19 asymptomatic cases underwent several MRI scans before surgery and mean diameter of asymptomatic tumors at scan closest to surgery was 32 mm and the median volume was 10.7 cm^3^ (see Fig. 2). In addition, radiological finding of edema was present in 40.0% of asymptomatic cases but in 66.7% of controls (*p* = 0.02). Among the control group, the most common symptom at onset of illness was symptoms related to intracranial pressure (e.g., headache and/or vomiting) in 42.4%, followed by seizure (37.8%), and motor deficit (24.4%). In the asymptomatic patients, the most common indication of surgery was growth (34.9%) followed by patient’s preference in 25.6%. For further details concerning baseline characteristics, see Table 2.

When predicting the risk of rapid growth, we found that 16 (35.6%) of patients received 0–2 points in the AIMSS which placed them in the low-risk group. Moreover, 18 (40%) and 11 (24.4%) patients were given scores that placed them in the intermediate- and high-risk groups, respectively.

### Clinical outcome

Table 3 summarizes the outcome variables. Time from diagnosis to surgery differed significantly between the two groups; the median time from diagnosis to surgery was 8.6 months among the asymptomatic cases and 1.3 months among the controls (*p* < 0.001).

**Table 3:** Outcome characteristics

Outcome variables	Case (*n* = 45)	Control (*n* = 45)	*p* value
Months from diagnosis to surgery, median (Q1–Q3)	8.6 (3.4–20.1)	1.3 (0.7–4.1)	< 0.001
ASA score			0.76
1, *n* (%)	22 (48.9)	19 (42.2)	
2, *n* (%)	17 (37.8)	21 (46.7)	
3, *n* (%)	6 (13.3)	5 (11.1)	
Complications within 30 days, total *n* (%)	16 (35.6)	11 (24.4)	< 0.001
Postoperative hematoma, *n* (%)	3 (6.7)	2 (4.4)	1.0
Infection*, *n* (%)	3 (6.7)	6 (13.3)	0.49
Seizure, *n* (%)	3 (6.7)	0 (0)	0.24
New/worsened focal deficit, *n* (%)	4 (8.9)	3 (6.7)	1.0
Significant edema, *n* (%)	1 (2.2)	0 (0)	1.0
Other, *n* (%)	2 (4.4)	0 (0)	0.49
Age > 70 and complication, *n* (% of total patients aged > 70)	3/8 (37.5)	1/8 (12.5)	< 0.001
Days from surgery to complication, median (Q1–Q3)	2 (0–4)	15 (0–20)	0.24
Reoperation due to complication, *n* (%)	3 (6.7)	7 (15.6)	0.32
Postoperative rehabilitation, *n* (%)	8 (18.6)	7 (16.3)	1.0
Course of disease > 1 year			0.11
Recurrence, *n* (%)	4 (9.5)	0 (0)	
Progress of remnant, *n* (%)	2 (4.8)	5 (11.4)	
Stable disease, *n* (%)	27 (60.0)	34 (75.6)	
Missing, *n* (%)	12 (26.7)	6 (13.3)	
Adjuvant therapy, *n* (%)	2 (4.4)	2 (4.4)	1.0
Missing, *n* (%)	1 (2.2)	0 (0)	
Reoperation of tumor, *n* (%)	3 (6.7)	4 (8.9)	0.21
Follow-up > 1 year			
Focal deficit, *n* (%)	4 (8.9)	7 (15.6)	0.63
Epilepsy/AED, *n* (%)	3 (7.7)	14 (32.6)	< 0.01
Missing, *n* (%)	6 (13.3)	2 (4.4)	
Postoperative work status			0.74
Pension, *n* (%)	13 (28.9)	13 (28.9)	
Sick leave, *n* (%)	2 (4.4)	4 (8.9)	
Partial sick leave, *n* (%)	3 (6.6)	5 (11.1)	
Working full hours, *n* (%)	16 (35.6)	13 (28.9)	
Other, *n* (%)	1 (2.2)	2 (4.4)	
Missing, *n* (%)	10 (22.2)	7 (15.6)	
Death before end of follow-up, *n* (%)	2 (4.4)	3 (6.7)	1.0

*Infections such as local wound infection, pneumonia, and urinary tract infection

AED was used by 32.6% of symptomatic patients at long-term follow-up postoperatively while 7.7% of the asymptomatic cases were on AED treatment (*p* < 0.01). Complications within 30 days after surgery differed significantly between the two groups; 16 (35.6%) of asymptomatic cases compared with 11 (24.4%) of the symptomatic controls (*p* < 0.001). Likewise, the proportion of patients 70 years of older experiencing complication(s) was higher in asymptomatic patients (3/8, 37.5% vs. 1/8, 12.5%; *p* < 0.001). Mean age among asymptomatic patients with postoperative complication was 64.1 (± 9.6). In the asymptomatic group, there were three patients (7.7%) with new onset seizure and four patients (8.9%) with new deficit postoperatively with implications also in the longer term (see Fig. 2). The frequency of reoperation due to complication was 15.6% in controls compared with 6.7% in asymptomatic cases (*p* = 0.32). The complication rate among the group of cases undergoing surgery due to patient preference was 45%, as compared with 36% in the group of meningioma cases that showed growth in volume on consecutive MRIs. Thirty-seven percent of the smaller tumors cases (volume smaller than median at time of diagnosis) had an adverse event compared with 38% of the larger tumors (volume larger than median at time of diagnosis).

Postoperatively the proportion of asymptomatic cases working full time had decreased from 46.3 to 35.6%. In the symptomatic controls, the proportion benefiting from sick pay and part-time work had increased from 4.4 to 20%.

Recurrence was observed in 9.5% of asymptomatic patients while none of the symptomatic controls had recurrence. However, progression was observed in 4.8% of the asymptomatic cases compared with 11.4% of the controls.

## Discussion

In this single-center case-control study, we have described the management and outcome of surgically treated meningioma compared with a symptomatic control group. The groups were similar at baseline with the exception of tumor size and symptoms. In the asymptomatic group, growth was the most common indication for surgery. Unsurprisingly, patients without symptoms waited longer prior to surgery compared with the symptomatic group. Finally, asymptomatic patients experienced similar outcome compared with the symptomatic ones, including a considerable morbidity even at the longer term.

Surgical resection has been reported to be beneficial in asymptomatic meningiomas in order to remove the tumor prior to growth that might make surgery more complicated [23]. This is perhaps looking at what is complicated from the surgeon perspective instead of taking the patient perspective of complications. We observed that a considerable group is operated due to growth alone without the development of symptoms. This is probably a decision based upon presumed future growth dynamics and later symptom development, but it may not be so straightforward to predict future growth based upon previous growth in meningiomas. A study published in 2011 by Nakasu et al. [16] reported that the growth of meningiomas fits an S-shaped curve (e.g., Gompertzian growth) better than the linear growth model, with the conclusion that meningiomas grow more slowly in elderly than younger patients, and dependent of tumor size. The same study also demonstrated that incidental meningiomas to a greater extent than their symptomatic counterparts reach their inflection point of slowed growth before diagnosis, which explains why their growth is slower and many remain asymptomatic for years. However, many elderly patients presenting with an asymptomatic medium size tumor have had a tumor that at some point increased in size, but the growth rate may have reached the inflection point of slowed growth prior to imaging diagnosis. Therefore, according to the results presented by Yoneoka et al. [25], the majority of patients with asymptomatic meningiomas can be actively followed without intervention, even if some growth occurs.

A recent review reported that among 15 included studies of actively monitored incidental meningiomas, the follow-up regimens varied widely [7–9, 12, 15, 18, 22]. In 2017, Lee et al. published a new scoring system (AIMSS) with the aim of estimating the risk for rapid growth of asymptomatic meningiomas [11]. According to radiological characteristics, the tumors are classified into low-, intermediate-, and high-risk groups for rapid growth and hence development of symptoms. In our population, 16 (35.6%) of patients were placed in the low-risk group, with predicted < 10% risk of rapid growth, thereby weakening the indication for surgical indication according to this scoring system. The scoring system does not take into account other clinical characteristics such as gender, age, tumor location, or tumor morphology, which have been found to be predictive of significant growth in asymptomatic meningiomas in other studies [11, 15, 25]. In a similar way, Islim et al. [6] present a prognostic model of disease progression developed from a retrospective cohort of 459 asymptomatic meningiomas. The patients were stratified based on imagining parameters such as tumor volume, tumor hyperintensity, peritumoral signal change, and proximity to critical neurovascular structures, and then placed into low-, medium- and high-risk groups. The study found that the 5-year disease progression rate was 3%, 28%, and 75%, respectively, and that the risk of disease progression plateaued after 5 years of follow-up in all groups. Thus, both scoring systems need to be further developed and validated; but might serve as a guide in the future to help physicians to tailor the periodicity of radiological and clinical controls.

We found that patients without symptoms waited significantly longer prior to surgery (8.6 months), and in approximately 35%, growth was demonstrated influencing the surgical indication. This finding corroborates a recently published Swedish registry-based study where a difference in waiting times was noted [3]. In the literature, the waiting time from diagnosis to intervention for asymptomatic meningiomas range from 7.3 to 48.8 months [3, 7, 9].

Most asymptomatic meningiomas are, and perhaps should be, monitored for some time before intervention. Histopathological grading often shows WHO grade I or II meningioma, but in our series, we found one anaplastic meningioma of WHO grade III. This has previously been reported in the literature, and the review by Islim et al. [7] presents that 3 out of 316 (0.95%) patients had a grade III meningioma. Hence, anaplastic meningiomas are very rare among asymptomatic meningiomas but still sometimes seen. This however does not validate resecting every asymptomatic meningioma. The grade III meningioma in question in our series showed consecutive growth over time, almost doubling in tumor diameter between first and last MRI preoperatively. Therefore, a period of monitoring will surely reveal growth in cases such as this, leading to intervention and correct histopathological diagnosis with following adjuvant treatment.

In our study population, a surprising higher complication rate compared with the symptomatic control group was found. This is in contrast to a previously published study which found an overall lower complication rate in asymptomatic meningiomas as compared with their symptomatic counterparts [26]. Yano et al. indicate that patients aged > 70 years have a higher complication rate as compared with younger patients (9.3% vs. 4.4%) [24]. In our study population, we found that the proportion of patients 70 years and older who developed postoperative complications was 37.5%. Although not apparent in our study, increased morbidity rate has been reported for patients older than 70 years when compared with their younger counterparts in studies with larger sample sizes [1, 10]. Finally, we observed that the preoperatively asymptomatic patients working full time decreased with 23% when evaluated more than 1 year after surgery as compared with preoperatively. Altogether, these figures give rise to some concern and must be considered prior to intervention in patients without symptoms. In this study, we make no attempt to present the management as the optimal clinical decision-making process, but we describe real-life decisions. We hope this can create further discussion on the topical issue of management of asymptomatic meningioma [6, 7, 23]. Now we can only speculate how nuanced counseling on pros and cons of surgery was provided to the 25.6% of the asymptomatic cases that were operated upon because of the patient’s own wish as main indication.

Based on our findings, we suggest a more restrictive approach, especially for the older patients with asymptomatic meningiomas. In this particular subgroup, patient’s preference and (slow) growth without symptoms are perhaps not reasonable indications for surgery. An incidentally found asymptomatic meningioma in an older individual can remain asymptomatic throughout the remainder of this patient’s life, despite minimal growth since inflection point of growth curve may have been reached or will be reached soon. Just as with other slow-growing tumors, such as tumors of the prostate gland, one might consider a concept of “watchful waiting” rather than “active monitoring” for the more elderly population [4, 14]. This means deferring from the belief that most tumors will eventually need surgical intervention, instead carefully follow the clinical development of potential symptoms and let that be the deciding factor for when surgery is appropriate. A prerequisite for the success of watchful waiting is well-educated elderly patients and relatives, to be able to capture symptoms that should not be assigned to increasing age only. Nevertheless, this approach requires further prospective studies to establish evidence-based and patient-safe guidelines tailored for patients with asymptomatic meningiomas.

## Limitations and missing data

Significant limitations of this study are the retrospective nature and relatively small number of patients. For certain variables, the amount of missing data was significant, and although change of medical journal systems and lack of storage of images in PACS were the main reasons, we cannot exclude that this has introduced some bias.

All reviews of medical records and volume measurements were made by one author (O.N.), and due to the lack of standardization with regard to how symptoms, complications, follow-up, and reasoning have been documented in the medical journals, some assumptions have had to be made which could contribute with some inaccuracies. Importantly, the matching was done without knowledge of the other clinical variables.

## Conclusions

We found that asymptomatic patients had smaller tumor and waited longer prior to surgery. The main indications for surgery were growth and patient’s wish. This needs to be balanced against the observation that complications in asymptomatic patients were as least as common as in symptomatic patients. Further, patients without symptoms from the meningioma preoperatively were less likely to work full time postoperatively as compared with preoperative status. Considering also the reports of the growth curve of asymptomatic meningiomas, it may be reasonable to suggest a more restrictive approach, especially in the elderly population.

## Electronic supplementary material

ESM 1 (DOCX 190 kb)

## Abbreviations

WHO: World Health Organization
EANO: European Association of Neuro-Oncology
MRI: Magnetic resonance imaging
AIMSS: Asian Intracranial Scoring System
NF: Neurofibromatosis
AED: Anti-epileptic drug
