# Supplementary material for: Indications and outcome in surgically treated asymptomatic meningiomas: a single-center case-control study
Source: Acta Neurochir (Wien). 2020 Feb 3;162(9):2155–63. doi: 10.1007/s00701-020-04244-6 (PMC7415028; doi:10.1007/s00701-020-04244-6)
Supplement: Supplementary file 1 — (DOCX 190 kb) [file 701_2020_4244_MOESM1_ESM.docx]

**Supplementary material**

***Figure 3:*** *Representative T2 axial slice from the diagnostic MRI examination for cases presenting with tumor adjacent brain edema. Left to right, patient 1 (upper left) through patient 45 (lower right).*

***
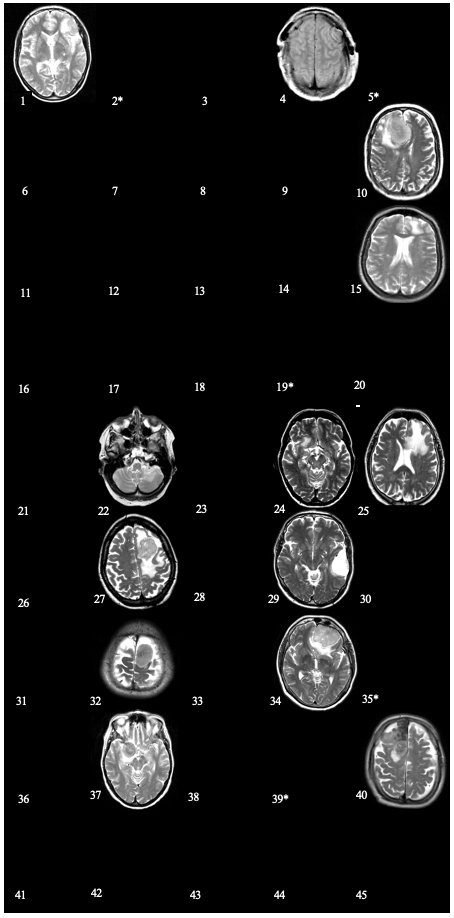
***

** Case with adjacent edema lacking representative T2 MRI*
